# Supplementary material for: A low-level Cdkn1c/p57kip2 expression in spinal progenitors drives the transition from proliferative to neurogenic modes of division
Source: EMBO Rep. 2025 Dec 8;27(2):433–70. doi: 10.1038/s44319-025-00653-9 (PMC12852696; doi:10.1038/s44319-025-00653-9)
Supplement: Supplementary file 1 — Table EV1 [file 44319_2025_653_MOESM1_ESM.docx]

**Table EV1**

Summary of the numbers of cells counted in figures 5A, 5D, 6A and 6B.

| **Figure 5A** | **Number of GFP/pRb double positive cells** | |
| --- | --- | --- |
|  | **CTRL sh** | **Cdkn1c sh** |
| 1hour | 916 | 730 |
| 4hours | 976 | 954 |
| 7hours | 1415 | 1846 |
| 10hours | 756 | 1254 |
| 12hours | 1072 | 1739 |
| 14hours | 702 | 1148 |
| 17hours | 689 | 802 |
| **Figure 5D** | **Number of FT/GFP/pRb triple positive cells** | |
|  | **CTRL sh** | **Cdkn1c sh** |
| 2h30 | 73 | 76 |
| 4h30 | 78 | 64 |
| 6h30 | 253 | 350 |
| 8h30 | 172 | 186 |
| 10h30 | 118 | 240 |
| **Figure 6A** | **Number of GFP positive cells** |  |
| CTRL sh | 791 |  |
| CCDN1 sh | 1179 |  |
| Cdkn1c sh | 884 |  |
| Cdkn1c and CCDN1 sh | 783 |  |
| **Figure 6B** | **Number of FT/GFP positive cells** | **Number of FT/GFP/pRb triple positive cells** |
| CTRL sh | 268 | 118 |
| CCDN1 sh | 195 | 68 |
| Cdkn1c sh | 273 | 145 |
| Cdkn1c and CCDN1 sh | 184 | 68 |
