# Supplementary material for: A low-level Cdkn1c/p57kip2 expression in spinal progenitors drives the transition from proliferative to neurogenic modes of division
Source: EMBO Rep. 2025 Dec 8;27(2):433–70. doi: 10.1038/s44319-025-00653-9 (PMC12852696; doi:10.1038/s44319-025-00653-9)
Supplement: Supplementary file 3 — Expanded View Figures [file 44319_2025_653_MOESM3_ESM.pdf]

Expanded View Figures

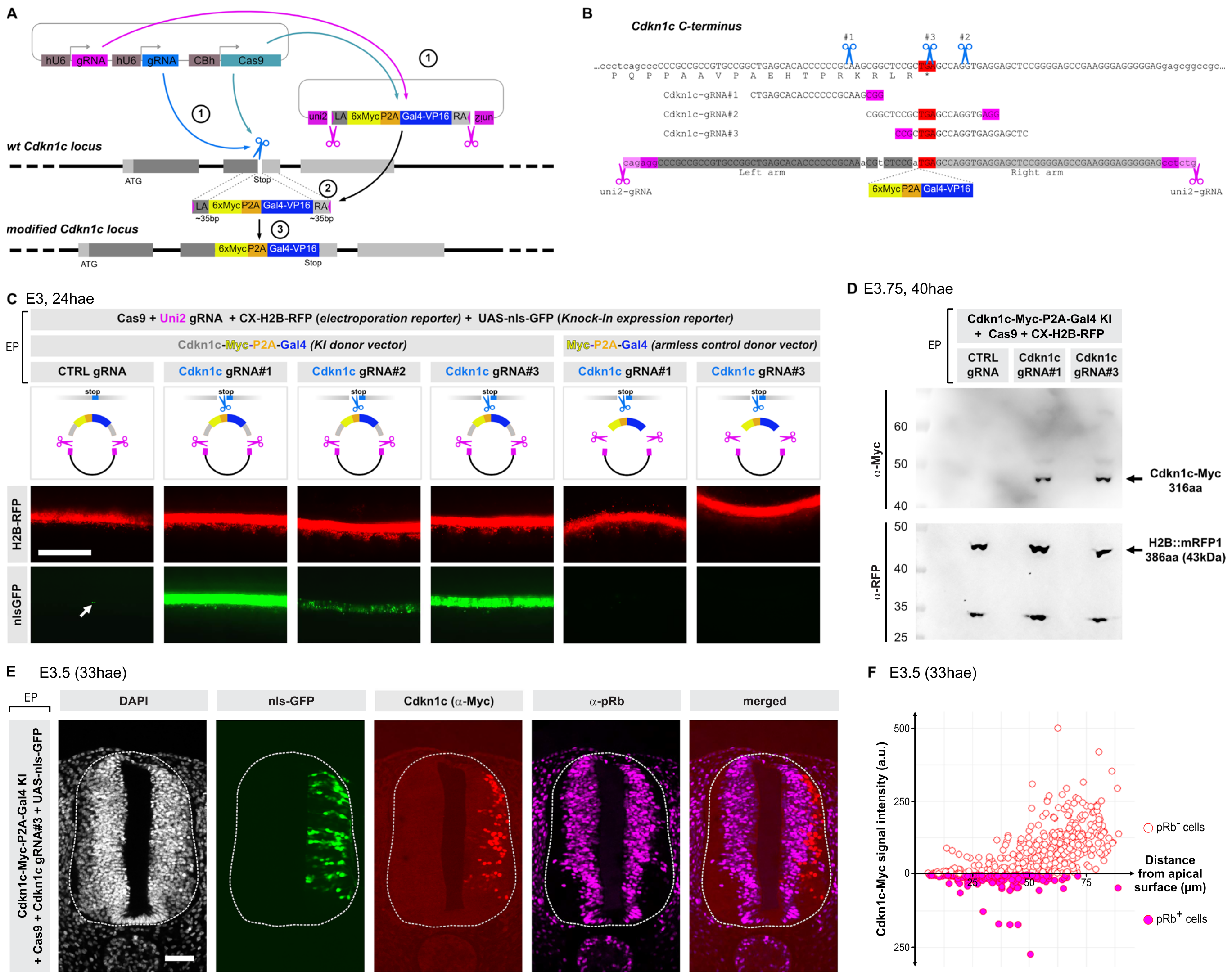

Figure EV1. Somatic knock-in strategy to target the endogenous *Cdkn1c* locus with Myc tags and monitor the dynamic expression of the protein in spinal cord progenitors.

(**A**) Microhomology Mediated End Joining (MMEJ) strategy used for the somatic knock-in. The donor plasmid carries short arms of homology (< 35 bp) to the *Cdkn1c* locus at the level of the C-terminus, flanking a sequence consisting of 6 Myc tags in frame with *Cdkn1c* coding sequence, a P2A pseudo cleavage sequence and the Gal4-VP16 synthetic transcription factor. This donor cassette is flanked on both sides by target sites for a "universal" guide RNA (uni2 gRNA) designed to trigger linearization of the vector and to release the donor cassette as a linear double-stranded DNA fragment in the electroporated cells. Somatic knock-in is achieved by the coelectroporation of a CRISPR/Cas9 vector that expresses the Cas9 protein, the uni2 gRNA targeting the donor vector for linearization, and the gRNA targeting the *Cdkn1c* locus. (**B**) Details of the targeted genomic sequence for Myc tagging of the endogenous *Cdkn1c* locus. Genomic sequence at the level of *Cdkn1c* C-terminus (top), sequence of the three gRNAs (middle), and sequence of the arms of homology used in the MMEJ construct (bottom, sequence highlighted in blue). The three bases highlighted in white represent silent base changes introduced in the left arm of homology to prevent recognition and cleavage of the donor vector by gRNA#1. Arrows indicate the theoretical cut sites of the three gRNAs on the target locus. (**C**) Validation of the efficiency and specificity of the knock-in strategy: the donor vector was co-electroporated with CRISPR/Cas9 vectors expressing either a control gRNA (CTRL gRNA) or one of the three gRNAs targeting the *Cdkn1c* locus. A UAS-nls-GFP vector was included in the electroporation mix to report expression of the Gal4-VP16 transcription factor in addition to an electroporation reporter (CX-H2B-mRFP). One representative embryo (out of 4–6) is shown for each condition, with similar electroporation level (red). gRNA#1 led to a strong GFP signal (green), showing the greatest efficiency. gRNA#3 appeared slightly less efficient, and gRNA#2 yielded a much lower signal. An "armless" construct lacking homology to the *Cdkn1c* locus was used as an additional negative control with gRNA#1 and gRNA#3. Specificity is demonstrated by the virtual absence of background GFP signal when the control gRNA or the armless donor vector are used (white arrow points to two GFP-positive cells or clusters of cells observed in the control gRNA embryo). Scale bar: 300 µm. (**D**) Western blot analyses on protein extracts from neural tubes electroporated with the *Cdkn1c-Myc* knock-in construct and control (CTRL) gRNA, Cdkn1c gRNA#1 or Cdkn1c gRNA#3, and a H2B-mRFP1 electroporation reporter construct. The anti-Myc antibody (top panel) reveals a strong and specific band with both gRNA#1 and #3, and no signal at all with a control gRNA. Of note, the ~45 kDa apparent size is higher than the expected ~35 kDa for the 316 aa long Cdkn1c-6xMyc fusion protein. This is consistent with previous observations with human and mouse Cdkn1c, which display a major band at 57 kDa in western blots despite a theoretical size of 35 kDa (Lee et al, 1995; Matsuoka et al, 1995). Bottom panel: detection of H2B-mRFP1 protein with an anti-RFP antibody was used as a loading control and reveals bands of similar intensity in all three conditions. (**E**) Endogenous Cdkn1c-Myc protein expression pattern at E3.5 (33 hae) using guide #3 to target *Cdkn1c* locus. Somatic knock-in of Myc tags at the *Cdkn1c* locus allows the visualization of Cdkn1c protein using an anti-Myc immunofluorescence (Myc, red) on transverse vibratome sections. Inclusion of a Gal4-VP16 transcription factor in the knock-in construct identifies all the cells which express or have previously expressed Cdkn1c via the UAS-nls-GFP reporter (green). Progenitor population was visualized by an anti-phospho-Rb antibody (pRb, magenta). The neural tube contour is highlighted by a dashed line. High levels of endogenous Cdkn1c-Myc signal was observed in the mantle zone contiguous to the ventricular zone while weaker Myc signal is observed in the progenitor zone. Scale bar: 50 µm. (**F**) Quantification of Cdkn1c-Myc expression in relationship with pRb expression along the apico-basal axis. Quantification of the Myc signal intensity from a *Cdkn1c-Myc* knock-in insertion was performed in vibratome sections from embryos electroporated with the donor vector, gRNA#3 and a UAS-nls-GFP reporter (**E**). Knock-in cells that express or have expressed Cdkn1c were identified on the basis of Myc and/or GFP positivity. The Myc signal intensity in individual is plotted on the *Y* axis towards the upper (pRb +) and lower (pRb-) parts of the graph as a function of their position along the apico-basal axis (X-axis). Data are from 558 cells from 12 sections from 5 embryos. hae: hours after electroporation.

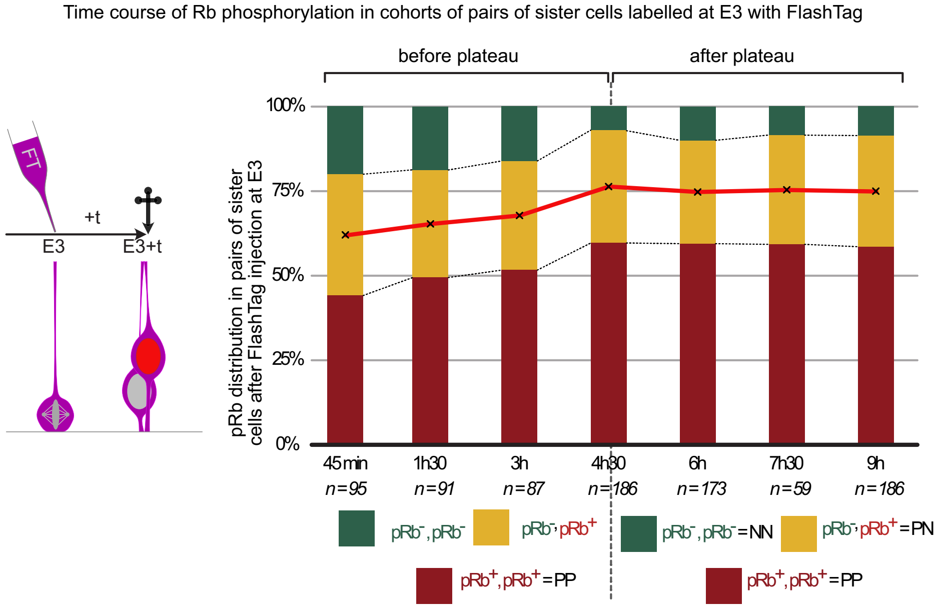

Figure EV2. pRb is a reliable marker of progenitor cells 6 h after mitosis.

Left: Experimental scheme for the determination of Rb phosphorylation status in FlashTag-labeled cells. Wild-type E3 embryos are injected with FlashTag (FT), which specifically labels cells synchronously undergoing mitosis at the time of injection. Embryos are collected at different timepoints (t) after injection to determine at which timepoint pRb positivity reaches a plateau in the cohort of FlashTag-positive daughter cells. Right: Time course of pRb expression in FlashTag-positive pairs of sister cells at consecutive time points after injection. FlashTag injection was performed at E3 and embryos were harvested at the indicated timepoints after injection. Thoracic vibratome sections were immunostained with anti-pRb antibody to evaluate the pRb status in pairs of FlashTag-positive sister cells. The proportion of pairs with two pRb-positive cells (red), one pRb-positive cell (yellow) or zero pRb-positive cell (green) becomes stable after 4h30. This indicates that the proportion of individual pRb-positive cells (red line) reaches a plateau corresponding to the proportion of cycling progenitors in this cohort. Therefore, after that time point, pRb positivity becomes a reliable marker of progenitor status in FlashTag labeled cells. This allows the retrospective attribution of the mode of division (PP, PN or NN) of the mother of pairs of sister cells via pRb labeling. $N = 2$ to 5 embryos per time point. The number of pairs analyzed at each time point is indicated at the bottom of the diagram.

 © The Author(s)

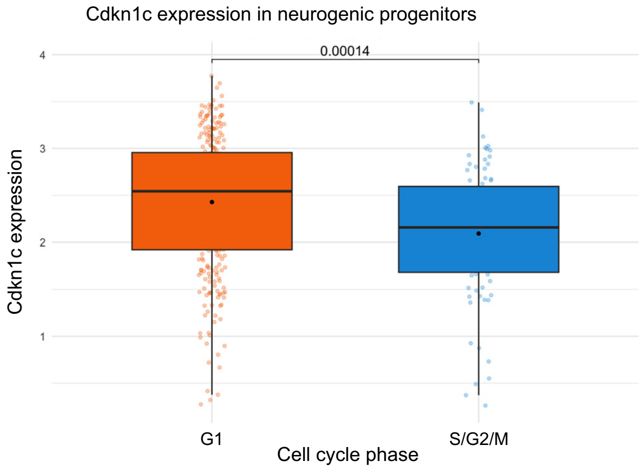

Figure EV3. Predominant expression of *Cdkn1c* transcript in the G1 phase of neural progenitors.

The "Neurogenic Progenitor" population identified in the scRNAseq dataset (362 cells from a single dataset) was split in two populations [G1 ($n = 282$ cells) and S/G2/M ($n = 80$ cells)] according to cell cycle scores. *Cdkn1c* mRNA expression was compared between these two populations, revealing a significantly higher expression in G1 compared to the other cell cycle phases. Statistical test: Wilcoxon test, $P = 0.00014$. Black dots: median; Horizontal bars: mean; box plot bounds: 1st and 3rd quartiles; whiskers: 1.5xIQR values from median.

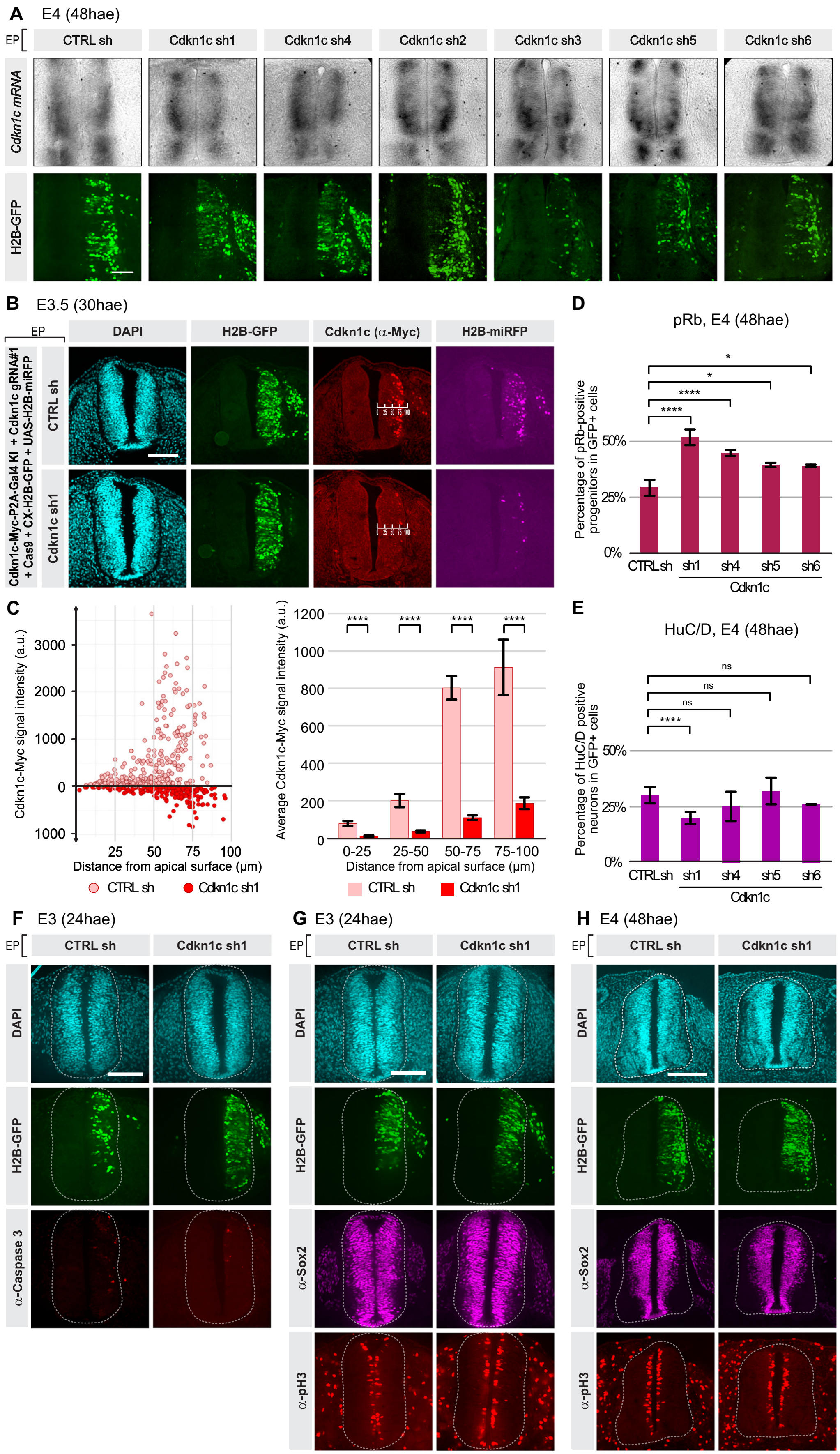
A E4 (48hae)
EP
CTRL sh
Cdkn1c sh1
Cdkn1c sh4
Cdkn1c sh2
Cdkn1c sh3
Cdkn1c sh5
Cdkn1c sh6
Cdkn1c mRNA
H2B-GFP
B E3.5 (30hae)
DAPI
H2B-GFP
Cdkn1c (α-Myc)
H2B-miRFP
Cdkn1c-Myc-P2A-Gal4 KI + Cdkn1c gRNA#1 + Cas9 + CX-H2B-GFP + UAS-H2B-miRFP
CTRL sh
Cdkn1c sh1
C
Cdkn1c-Myc signal intensity (a.u.)
Distance from apical surface (μm)
CTRL sh
Cdkn1c sh1
Average Cdkn1c-Myc signal intensity (a.u.)
0-25
25-50
50-75
75-100
D
pRb, E4 (48hae)
Percentage of pRb-positive progenitors in GFP+ cells
CTRL sh
sh1
sh4
sh5
sh6
Cdkn1c
E
HuC/D, E4 (48hae)
Percentage of HuC/D positive neurons in GFP+ cells
F E3 (24hae)
G E3 (24hae)
H E4 (48hae)
α-Caspase 3
α-Sox2
α-pH3

 © The Author(s)

Figure EV4. Partial knock down of *Cdkn1c* expression in spinal neural progenitors and prospective neurons via shRNA delays neurogenesis.

(**A**) mRNA expression of *Cdkn1c* in chick embryonic neural tube after electroporation of each of the six shRNAs. In situ hybridization and GFP immunofluorescence on the same cryosection of thoracic region of chick embryo. Upper panel: visible downregulation of *Cdkn1c* mRNA was only observed with shRNA1 (sh1) and to a lesser extent shRNA4 (sh4) conditions, while comparable mRNA expression to the control condition (CTRL sh) was observed with the other shRNAs (sh2,3,5,6) (compare left versus right hemitubes). Lower panel: Corresponding level of electroporation for each embryo (GFP immunofluorescence). Scale bar: 50 µm. (**B**) Endogenous Cdkn1c-Myc protein expression pattern at E3,25 (30hae) using guide #1 to target the *Cdkn1c* locus upon downregulation of *Cdkn1c* via shRNA approach. Top row: Control sh, bottom row: Cdkn1c sh. Somatic knock-in of Myc tags at the *Cdkn1c* locus allows the visualization of Cdkn1c protein using an anti-Myc immunofluorescence (Myc, red) on E3,25 transverse vibratome sections. A H2B-GFP construct (green) was used as a control of electroporation. Cells with a knock-in event were visualized thanks to the coelectroporation of a UAS:H2B miRFP construct (magenta). Scale bar: 100 µm. The graduated scales in the third column illustrate the bins used for quantification in (**C**). (**C**) Quantification of Cdkn1c-Myc expression along the apico-basal axis upon downregulation of *Cdkn1c* via shRNA approach. Quantifications of the Cdkn1c-Myc signal intensity from knock-in insertions were performed in vibratome sections from embryos electroporated with a control shRNA or Cdkn1c shRNA1 (see representative examples in (**B**)). Left graph: the Myc signal intensity measured in individual cells is plotted on the Y-axis towards the upper (CTRL sh) and lower (Cdkn1c sh1) parts of the graph as a function of their position along the apico-basal axis (X-axis). Right graph: bars represent the average Myc signal intensity in 25µm-wide bins along the apico-basal axis. CTRL sh: 347 cells from 5 embryos; Cdkn1c sh1: 233 cells from 5 embryos. Error bars show means ± SD; ****$P < 0.0001$ (Kolmogorov-Smirnov test). (**D**) Distribution of pRb-positive progenitors (red) in Cdkn1c shRNAs 1, 4, 5, 6 or control shRNA conditions at E4, 48 hae. (values for CTRL sh and Cdkn1c sh1 are identical to Fig. 3E). Error bars show means ± SD. CTRL vs sh1 and CTRL vs sh4, ****$P < 0.0001$; CTRL vs sh5 and CTRL vs sh6, *$P = 0.0365$; (unpaired Student's t test relative to CTRL sh). Numbers of counted electroporated cells: sh4, 3111 cells from 8 embryos; sh5, 1552 from 3 embryos; sh6, 931 cells from 3 embryos were analyzed. (**E**) Distribution of the HuC/D-positive neurons (magenta) in shRNAs 1, 4, 5, 6 or control conditions at E4, 48 hae. (values for CTRL sh and Cdkn1c sh1 are identical to Fig. 3F). Error bars show means ± SD; CTRL vs sh1, ****$P < 0.0001$. All others: ns, $P > 0.05$; (unpaired Student's *t* test relative to CTRL sh). Numbers of counted electroporated cells: sh4, 3111 cells from 8 embryos; sh5, 1552 from 3 embryos; sh6, 931 cells from 3 embryos were analyzed. (**F**) Cdkn1c knock-down did not induce cell death. Transverse vibratome sections of embryos 24 hae with a CTRL sh (left column) or Cdkn1c sh1 (right column). Immunostaining with an anti-Caspase3 antibody (red, 3rd row) show a signal in a few cells on the electroporated side (H2B-GFP, 2nd row), which is similar in both conditions. Top row: DAPI staining. Scale bar: 100 µm. (**G**, **H**) Cdkn1c knock-down did not induce ectopic localization of progenitors in the mantle zone. Transverse vibratome sections of embryos 24 hae (E3, (**E**)) or 48 hae (E4, (**F**)) with a CTRL sh (left column) or Cdkn1c sh1 (right column). Electroporated cells are marked with H2B-GFP (green, 2nd row). Immunostaining with an anti-Sox2 antibody (magenta, 3rd row) was restricted to the ventricular zone, and no ectopic cells were observed in the mantle zone. Similarly, mitotic figures labeled with an anti-pH3 antibody (red, 4th row) were restricted to the apical surface. Scale bars: 100 µm. hae hours after electroporation, CTRL control, sh shRNA.

© The Author(s)

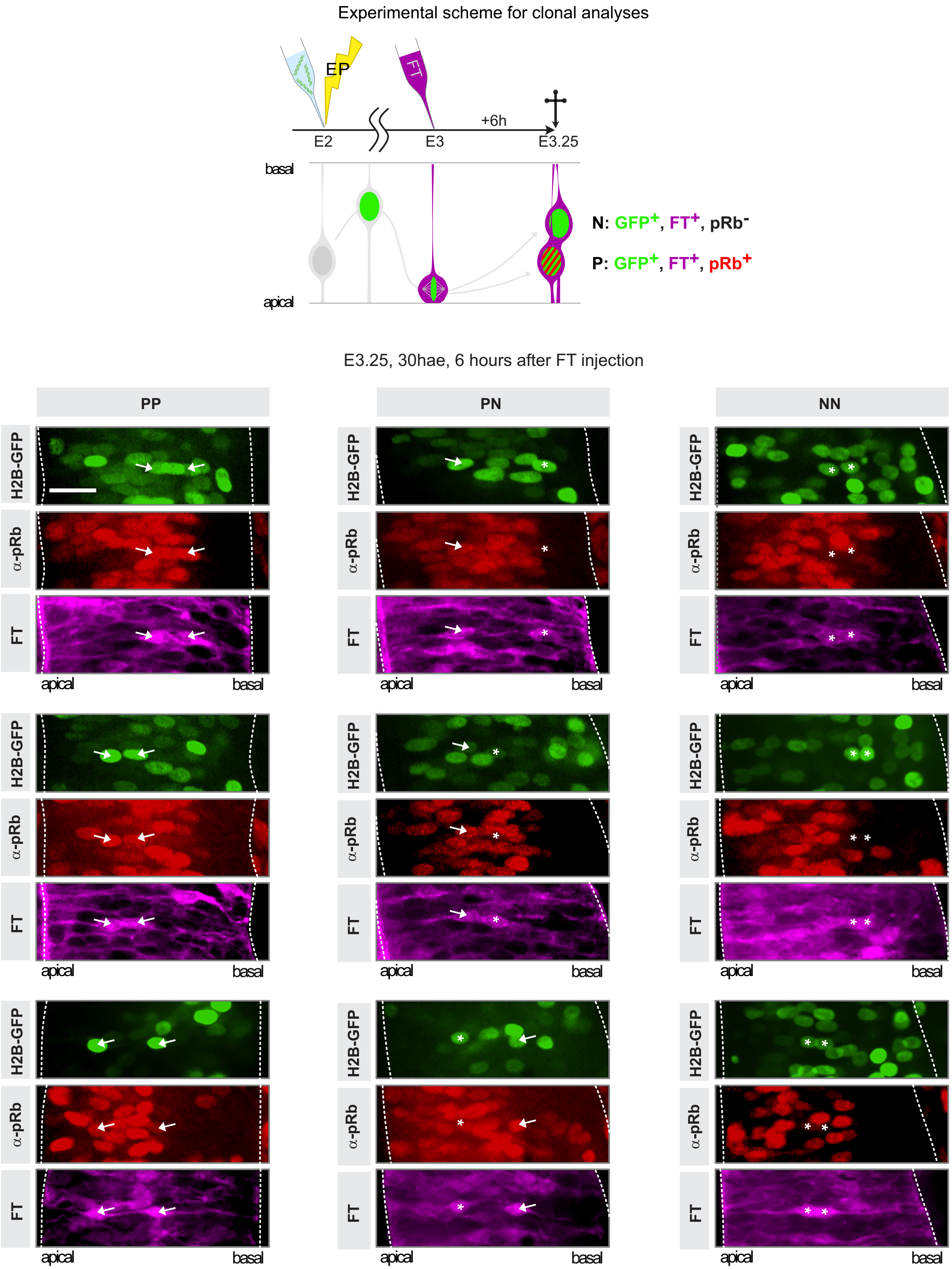
Experimental scheme for clonal analyses
EP
FT
+6h
E2
E3
E3.25
basal
apical
N: GFP+, FT+, pRb-
P: GFP+, FT+, pRb+
E3.25, 30hae, 6 hours after FT injection
PP
PN
NN
H2B-GFP
α-pRb
FT
apical
basal

 © The Author(s)

◀ **Figure EV5. FlashTag-based assay for the analysis of pairs of sister cells: principle and examples.**

Top: principle of the analysis of pairs of sister cells. Embryos are electroporated at HH13-14 (top left, yellow thunder) with Cdkn1c or control shRNA plasmids co-expressing a H2B-GFP reporter. Embryos are injected with the FlashTag dye (FT) 24 h after electroporation to label a synchronous cohort of mitotic progenitors, and collected 6 h later. At this time point after FT injection, anti-pRb immunofluorescence on thoracic vibratome sections determines the progenitor (pRb-positive) or neuron (pRb negative) status of electroporated (GFP-positive) pairs of FlashTag-positive sister cells (this panel is identical to Fig. 3G). Bottom: representative examples of two cell clones used for the quantification of modes of division depicted in Fig. 3H. Left to right panels show 3 examples each of PP pairs (column 1), PN pairs (column 2) and NN pairs (column 3). Arrows show pRb-positive (red) progenitors and asterisks show pRb negative neurons in FlashTag-positive (magenta) pairs of GFP-positive (green) sister cells. Scale bars: 25 µm.

© The Author(s)

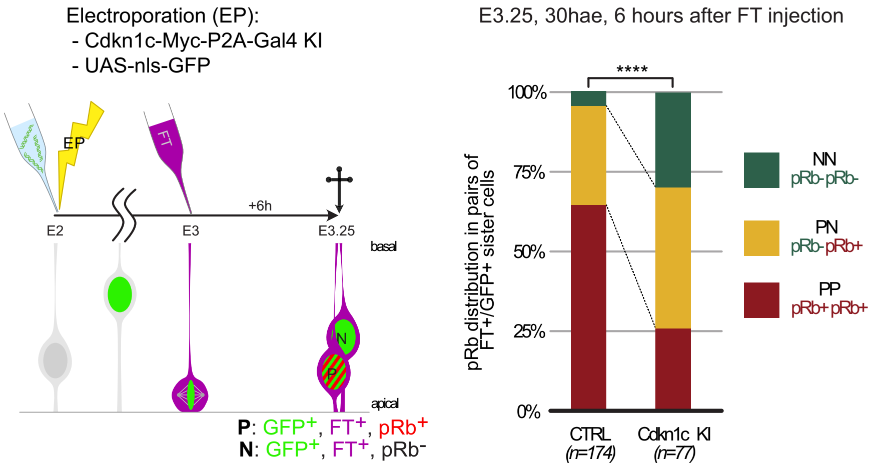

Figure EV6. Cdkn1c positive progenitors are more neurogenic than the overall progenitor population.

Left: Experimental scheme for the clonal analysis. A knock-in of the Gal4 reporter in the *Cdkn1c* locus was performed via in ovo electroporation (yellow thunder) at E2 and FlashTag (FT) was injected 24 h later. Sister cells born from Cdkn1c-positive progenitors dividing at the time of FlashTag injection were identified on the basis of the expression of a UAS-nls-GFP reporter and FlashTag positivity. Right: The distribution of PP, PN, and NN pairs is significantly different between *Cdkn1c* knock-in progenitors and FT-positive pairs of sister cells on the contralateral side of the same transverse vibratome sections, indicating that the Cdkn1c-positive population of progenitors is more neurogenic than the whole population at that stage. Statistical test: Chi-square test, *****P* < 0.0001; Chi-square value = 18.57. Pairs were obtained from 5 embryos. The number of pairs analyzed for both populations is indicated at the bottom of the graph.

 © The Author(s)

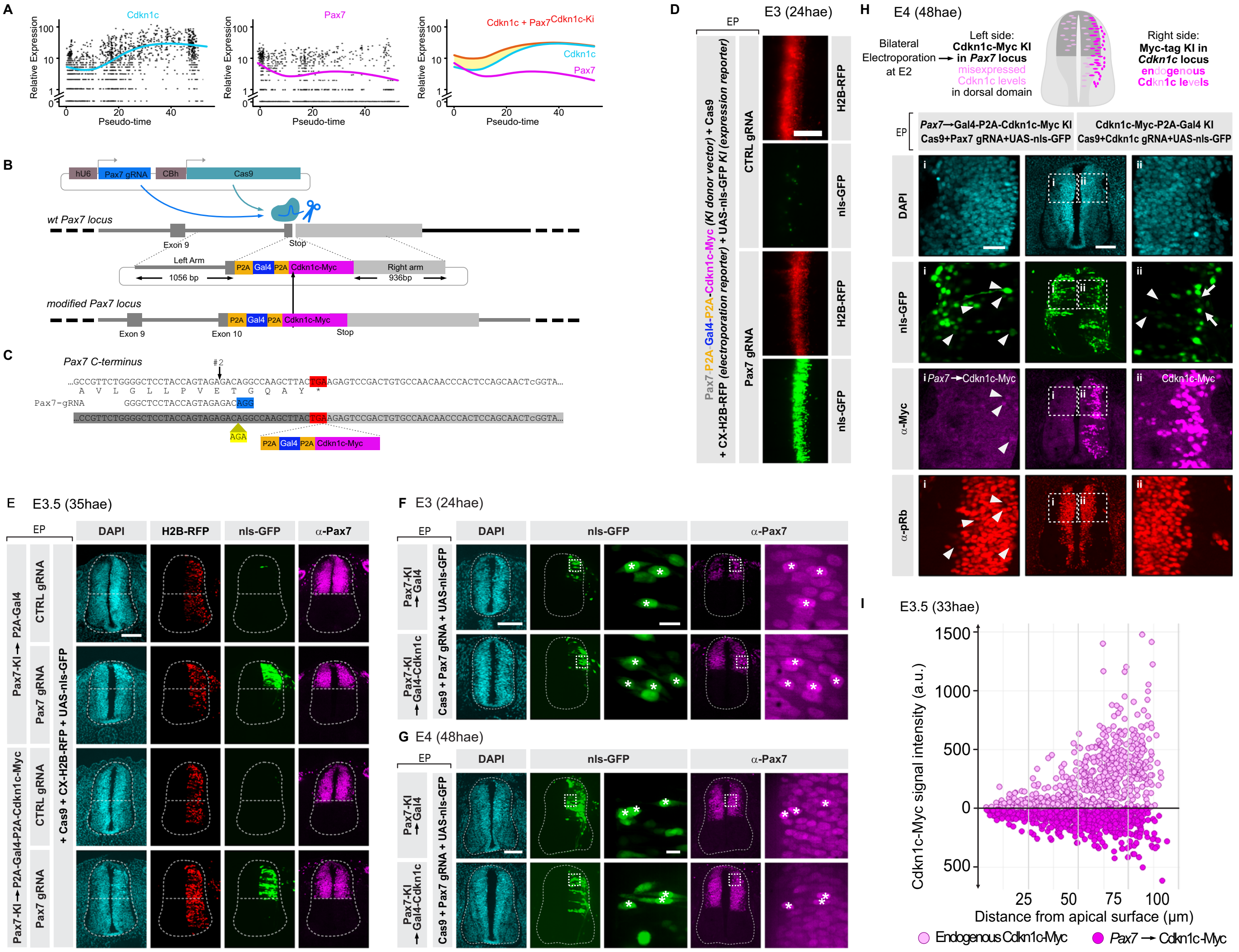
A
Cdkn1c
Pax7
Cdkn1c + Pax7
Relative Expression
Pseudo-time
B
hU6
Pax7 gRNA
CBh
Cas9
wt Pax7 locus
Exon 9
Stop
Left Arm
1056 bp
P2A
Gal4
Cdkn1c-Myc
Right arm
936bp
modified Pax7 locus
Exon 10
C
Pax7 C-terminus
Pax7-gRNA
AGA
D
E3 (24hae)
EP
CTRL gRNA
Pax7 gRNA
H2B-RFP
nls-GFP
E
E3.5 (35hae)
DAPI
H2B-RFP
nls-GFP
α-Pax7
F
E3 (24hae)
G
E4 (48hae)
H
E4 (48hae)
Bilateral Electroporation at E2
Left side: Cdkn1c-Myc KI in Pax7 locus
misexpressed Cdkn1c levels in dorsal domain
Right side: Myc-tag KI in Cdkn1c locus
endogenous Cdkn1c levels
α-Myc
α-pRb
I
E3.5 (33hae)
Cdkn1c-Myc signal intensity (a.u.)
Distance from apical surface (µm)
Endogenous Cdkn1c-Myc
Pax7 → Cdkn1c-Myc

Figure EV7. *Cdkn1c* knock-in at the *Pax7* locus leads to a premature low-level expression of Cdkn1c in dorsal neural progenitors.

(**A**) Expression levels of *Cdkn1c* and *Pax7* transcripts along the pseudo-time axis from the chick scRNASeq analysis. *Cdkn1c* expression has very low levels in "early" progenitors (left part of the pseudotime axis) and increases in more mature progenitors, before peaking in differentiating neurons. Pax 7 expression levels in "early" progenitors are slightly higher than those of *Cdkn1c*. The right panel shows an estimated cumulative expression (red) of endogenous (blue) and *Pax7*-driven (magenta) *Cdkn1c* levels upon knock-in of *Cdkn1c* coding sequences in the *Pax7* locus, which should result in premature expression in early progenitors, but no overexpression in newborn neurons. The scale for "Relative Expression" is logarithmic. (**B**) Principle of the "homology directed repair" (HDR) strategy used to drive low-level misexpression of *Cdkn1c*-Myc and Gal4-VP16 in dorsal progenitors from the *Pax7* locus. Somatic knock-in is based on a donor plasmid which carries long arms of homology (~1Kb) to the *Pax7* locus at the level of the C-terminus in exon 10. The arms of homology flank an "in-frame" knock-in cassette that consists in a P2A pseudocleavage site, the Gal4-VP16 synthetic transcription factor, a second P2A pseudocleavage site and the *Cdkn1c* coding sequence fused to three C-terminal Myc tags. This knock-in approach requires in addition the electroporation of a second vector expressing the Cas9 protein and a gRNA that targets the genomic region of *Pax7* upstream of the stop codon. Upon successful knock-in insertion, *Pax7* (gray), *Gal4-VP16* (blue) and *Cdkn1c-Myc* (magenta) coding sequences will be transcribed from the *Pax7* locus and co-translated. The insertion of P2A pseudocleavage sites (orange) between the three sequences will ensure that all three proteins are present as independent proteins. (**C**) Details of the targeted genomic sequence at the C-terminus of the *Pax7* locus. Genomic sequence at the level of *Pax7* C-terminus (top), sequence of the validated gRNA (Petit-Vargas et al, 2024) targeting this region (middle), and sequence of the arms of homology surrounding the knock-in cassette (bottom). To avoid possible targeting of the donor arms by the Pax7 gRNA, three bases (highlighted in yellow) were inserted 5 amino acids upstream of the *Pax7* stop codon. Note that this introduces an Arginine residue (AGA) in the Pax7 sequence. (**D**) Validation of the efficiency and specificity of the knock-in strategy. Imaging of the neural tube directly *in ovo*. The donor vector was co-electroporated together with a dual vector expressing the Cas9 nuclease and either a control gRNA (CTRL gRNA) or the gRNA targeting the *Pax7* locus. A UAS-nls-GFP vector was included in the electroporation mix to report expression of the Gal4-VP16 transcription factor. Finally, an electroporation reporter (CX-H2B-mRFP) was added to monitor the quality of electroporation. One representative embryo is shown for each condition, with similar electroporation level (red). Specificity is demonstrated by the virtual absence of background GFP signal when the control gRNA is used (compared to the massive GFP signal with Pax7 gRNA, only few GFP-positive cells are observed in the control embryo). Scale bar, 300 µm. (**E**) Transverse vibratome sections of embryos after knock-in of P2A-Gal4-VP16 somatic knock-in (top row) and P2A-Cdkn1c-P2A-Gal4-VP16 knock-in (bottom row) donor constructs at the *Pax7* locus at E3.5 (35hae). The UAS-nls-GFP signal (green) reports knock-in events and expression of Gal4-VP16 from the *Pax7* locus, and accordingly is restricted to the Pax7-domain (α-Pax7, magenta) in the dorsal half of the neural tube, and only in the presence of the Pax7-specific gRNA, despite electroporation all along the dorso-ventral axis, as revealed by the electroporation reporter H2B-RFP. The dashed line outlines the neural tube and the ventral limit of the Pax7 expression domain. Scale bar: 50 µm. (**F**, **G**) Characterization of Pax7 protein expression after knock-in at the *Pax7* locus. Immunostainings of Pax7 protein after somatic Knock-in of P2A-Gal4-VP16 (top row) or P2A-Gal4-VP16-P2A-Cdkn1c (bottom row) coding sequences at the *Pax7* locus on transverse vibratome sections of embryos 24 hae (E3, (**A**)) and 48 hae (E4, (**B**)). Knock-in events are identified via the co-electroporated UAS-nls-GFP reporter (green, second and third columns). First row: DAPI staining. Third and fifth rows of the panel show a close-up of the region highlighted by a dashed rectangle in the second and fourth row. A similar modest increase in Pax7 immunofluorescence is observed in knock-in cells (green, asterisks) in both conditions (compare top and bottom rows). Since this increase occurs irrespective of whether Cdkn1c is overexpressed or not, it does not affect the interpretation of phenotypes comparing the two conditions. The contour of the neural tube is underlined by a dashed line. Scale bars: 100 µm for first, second and fourth rows, 10 µm for close-ups. (**H**) *Pax7*-driven exogenous expression of Cdkn1c in dorsal progenitors mimics the levels of Cdkn1c expression in neurogenic progenitors at E4 (48 hae). A bilateral electroporation scheme was used to compare *Pax7*-driven levels of Cdkn1c expression (electroporation 1, left side hemi-tube, knock-in of Cdkn1c-Myc in the *Pax7* locus) with endogenous Cdkn1c levels (electroporation 2, right side hemi-tube, knock-in of a Myc tag in the *Cdkn1c* locus). The level of Cdkn1c-Myc (magenta) expression driven by *Pax7* is low and restricted to the ventricular region, where it is comparable to the endogenous levels of Cdkn1c-Myc expression in the contralateral side (i and ii, arrowheads). Note that although very few cells with a detectable Cdkn1c-Myc expression are observed in the misexpressed condition, the UAS-nls-GFP reporter is widely expressed, indicating strong electroporation and knock-in efficiency. This strong GFP signal, compared to the weak Myc signal, is explained by a differential stability and posttranslational regulation between Cdkn1c-Myc and Gal4-VP16, and by amplification of the GFP fluorescence via the Gal4/UAS system. Scale bars, 100 µm in central column and 30 µm in close-ups. (**I**) Quantification of endogenous versus *Pax7*-driven Cdkn1c-Myc expression along the apico-basal axis. The same vector combinations used in (**F**, **G**) were electroporated in separate sets of embryos to generate either a knock-in of the Myc reporter in the Cdkn1c locus reporting the endogenous expression level, or a knock-in of the Cdkn1c-Myc fusion in the *Pax7* locus, reporting the misexpression level. Quantifications of the Cdkn1c-Myc signal intensity from both conditions were performed 33 h after electroporation in vibratome sections. The Myc signal intensity measured in individual cells is plotted on the Y-axis towards the upper (endogenous Cdkn1c-Myc, light plum) and lower (*Pax7*-driven misexpressed Cdkn1c-Myc, magenta) parts of the graph as a function of their position along the apico-basal axis (X-axis). Endogenous Cdkn1c-Myc: 697 cells from 5 embryos; *Pax7*-driven Cdkn1c-Myc: 761 cells from 4 embryos. hae: hours after electroporation.

 © The Author(s)

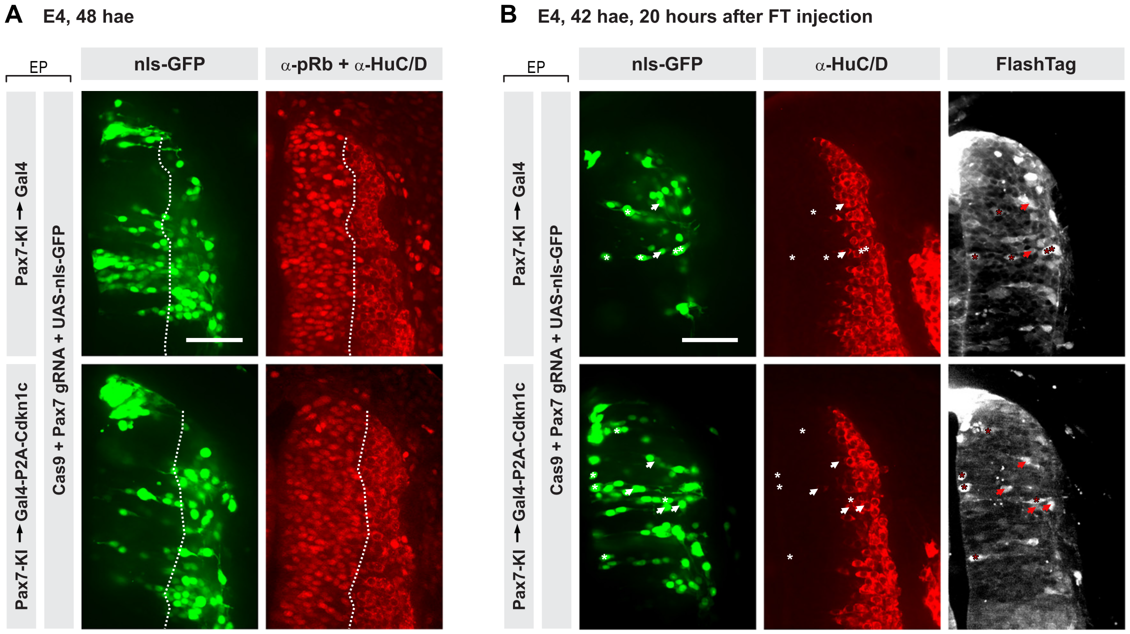

Figure EV8. Premature low-level expression of Cdkn1c in dorsal neural progenitors leads to accelerated neurogenesis.

(**A**) Transverse vibratome sections of embryos 48 h after electroporation of P2A-Gal4-VP16 somatic knock-in (top row) or P2A-Gal4-VP16-P2A-Cdkn1c knock-in (bottom row) constructs at *Pax7* locus. pRb and HuC/D immunofluorescence can be used to identify the nature of the cells (progenitor, pRb-positive nuclear signal in the ventricular zone or neuron, HuC/D-positive cytoplasmic signal in the mantle zone) that have undergone a knock-in event (UAS-nls-GFP, green signal). The dashed line marks the ventricular zone. Scale bar: 50 µm. (**B**) Transverse vibratome sections of embryos 42 h after electroporation of P2A-Gal4-VP16 somatic knock-in (top row) or P2A-Cdkn1c-P2A-Gal4-VP16 knock-in (bottom row) constructs at *Pax7* locus. FlashTag (gray) was injected 22 h after electroporation to label a cohort of mitotic cells, and embryos were collected 20 h later. HuC/D immunofluorescence (red) was used to identify neurons. Arrows point to triple positive (FlashTag +, GFP +, HuC/D +) cells and asterisks highlight double-positive (FlashTag +, GFP +, HuC/D−) cells. Scale bar: 50 µm. hae hours after electroporation.

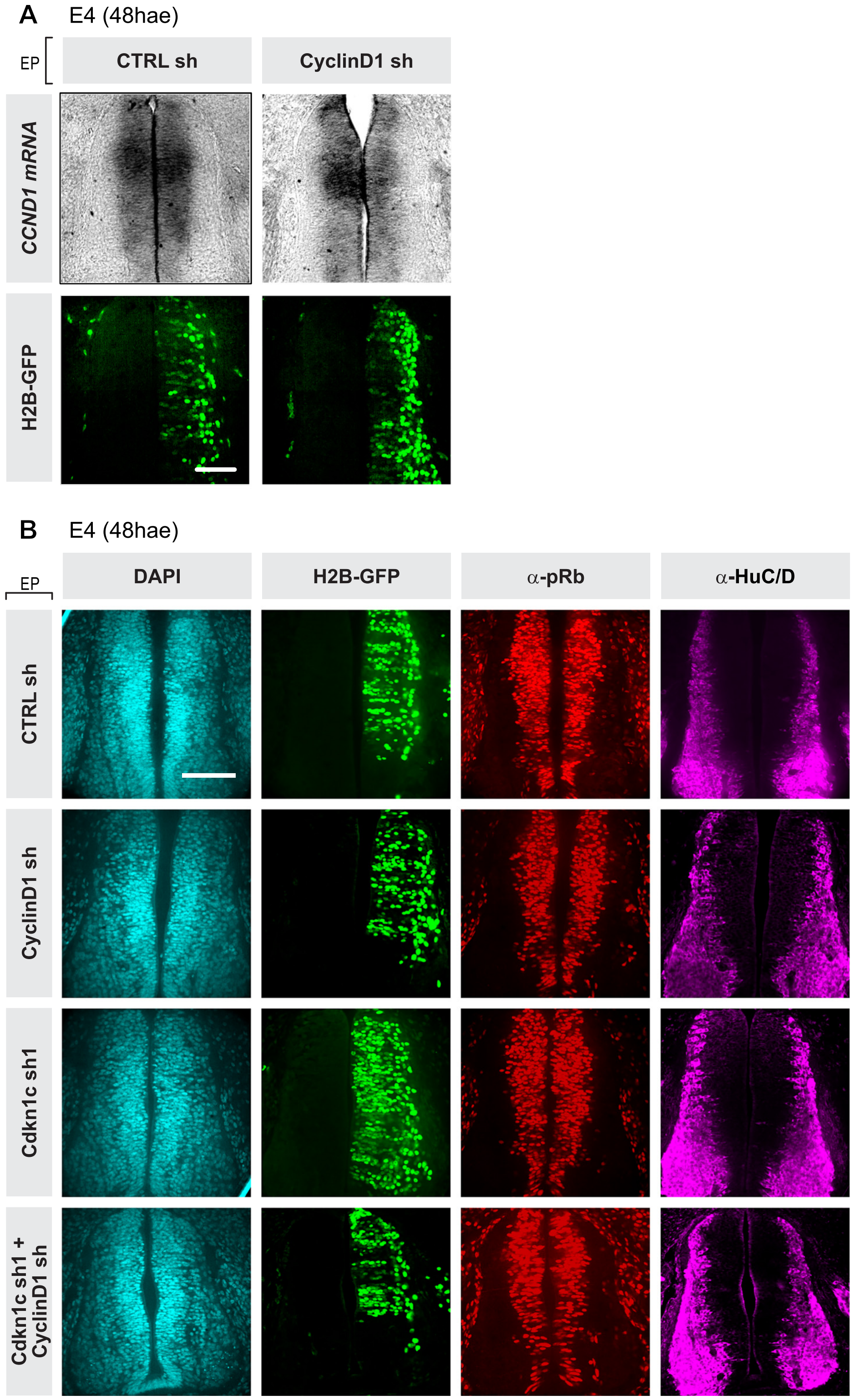

Figure EV9. Knock-down of *CyclinD1* mRNA rescues the Cdkn1c anti-neurogenic phenotype in the chick embryonic neural tube.

(**A**) In situ hybridization of a *CyclinD1* antisense probe on transverse cryosections of chick embryonic neural tube followed by an anti-GFP immunostaining to reveal electroporated cells two days (E4, 48 hae) after CyclinD1 shRNA (CyclinD1sh) electroporation. Scale bars: 100 μm. (**B**) Transverse vibratome sections of the chick neural tube (thoracic level) at E4 (HH st22) stained with HuC/D antibody (magenta) to label neurons and anti-pRb (red) antibody to label progenitors in control, single CyclinD1, single Cdkn1c or double CyclinD1/Cdkn1c shRNAs (sh) conditions. Representative images related to the quantification of the percentage of pRb-positive and HuC/D-positive cells within the electroporated population (GFP-positive) shown in Fig. 6D,E. Scale bar: 100 μm. hae: hours after electroporation.

 © The Author(s)
